# Prevalence and Predictors of Hypercoagulable Disorders in Patients With Concomitant Acute Limb Ischemia and Ischemic Stroke: A Cross-Sectional Study

**DOI:** 10.7759/cureus.115394

**Published:** 2026-08-29

**Authors:** Md. Aminul Islam, Shuvro Saha, Md. Mazharul Islam, Kazi Irfan Subhan, Gazi M. Habibullah

**Affiliations:** 1 Department of Neurology, Dhaka Medical College and Hospital, Dhaka, BGD; 2 Department of Neurosurgery, Dhaka Medical College and Hospital, Dhaka, BGD; 3 Department of Neurophysiology, National Institute of Neurosciences & Hospital, Dhaka, BGD; 4 Department of Neurosurgery, Rajshahi Medical College, Dhaka, BGD

**Keywords:** acute limb ischemia, antiphospholipid syndrome, arterial thrombosis, hypercoagulable state, ischemic stroke

## Abstract

Background

Acute limb ischemia (ALI) and ischemic stroke are serious arterial thrombotic presentations. When they occur in the same patient, particularly without an adequate conventional explanation, an underlying systemic thrombotic tendency may warrant investigation. However, evidence describing the frequency and clinical profile of hypercoagulable disorders in patients with concomitant ALI and ischemic stroke remains limited.

Objective

To determine the prevalence and spectrum of inherited and acquired hypercoagulable disorders among adults presenting with concomitant ALI and ischemic stroke and to identify clinical factors independently associated with a hypercoagulable state.

Methods

This prospective, hospital-based cross-sectional observational study was conducted in the Department of Neurology of a tertiary care teaching hospital in Bangladesh from January 2022 through December 2025. One hundred consecutive adults with objectively confirmed ALI and imaging-confirmed ischemic stroke were enrolled. Laboratory assessment included selected inherited and acquired thrombophilic abnormalities. Multivariable logistic regression was used to identify independent clinical factors associated with a hypercoagulable disorder.

Results

The study included 100 patients with a mean age of 56.8 ± 11.4 years, of whom 62 (62.0%) were female subjects. At least one selected hypercoagulable or prothrombotic abnormality was identified in 39 (39.0%) patients. Antiphospholipid syndrome (APS) was the most frequently identified abnormality, occurring in 18 (18.0%) patients, followed by hyperhomocysteinemia in 12 (12.0%), protein S deficiency in two (2.0%), protein C deficiency in three (3.0%), antithrombin III deficiency in two (2.0%), factor V Leiden mutation in three (3.0%), and prothrombin G20210A mutation in one (1.0%). Patients with a selected hypercoagulable or prothrombotic abnormality were younger than those without one (53.7 ± 9.8 vs. 58.8 ± 11.2 years; p=0.019) and were more frequently female (29/39 (74.4%) vs. 33/61 (54.1%); p=0.041). Previous ischemic stroke, previous venous thromboembolism (VTE), and Rutherford class IIb/III ALI were also more frequent among patients with a selected abnormality. Rutherford class IIb/III ALI was present in 27/39 (69.2%) patients with a selected abnormality compared with 20/61 (32.8%) patients without one (OR, 4.61; 95% CI, 1.94-10.95; p<0.001). In multivariable analysis, age <60 years (adjusted OR, 2.84; 95% CI, 1.16-6.91; p=0.022), female sex (adjusted OR, 2.18; 95% CI, 1.01-4.74; p=0.047), previous VTE (adjusted OR, 3.91; 95% CI, 1.29-11.84; p=0.016), and Rutherford class IIb/III ALI (adjusted OR, 2.86; 95% CI, 1.15-7.10; p=0.024) were independently associated with the presence of a selected hypercoagulable or prothrombotic abnormality.

Conclusion

Hypercoagulable abnormalities were identified in 39 (39.0%) patients presenting with concomitant ALI and ischemic stroke, with APS being the most frequently identified abnormality. Younger age, female sex, previous VTE, and more severe ALI were independently associated with a hypercoagulable state. These findings support selective thrombophilia evaluation in appropriately selected patients rather than routine testing of all patients with arterial thrombosis.

## Introduction

Acute limb ischemia (ALI) represents a sudden deterioration in arterial blood flow that can rapidly jeopardize limb viability, making prompt diagnosis and treatment essential. Despite improvements in revascularization techniques and supportive management, patients with ALI continue to experience considerable risks of limb loss, mortality, and hospital readmission [1]. Ischemic stroke likewise represents a major arterial ischemic disorder and is a leading contributor to death and persistent neurological disability worldwide [2]. Although cerebral and peripheral arterial ischemic events usually arise from more common mechanisms such as atherosclerosis or cardioembolism, their occurrence in the same patient is uncommon and may raise suspicion of an underlying systemic prothrombotic tendency [3,4].

Thrombophilic conditions comprise a heterogeneous group of acquired and inherited disorders. Acquired abnormalities include antiphospholipid syndrome (APS), whereas inherited conditions may involve deficiencies or abnormalities of protein C, protein S, antithrombin, Factor V Leiden, or the prothrombin G20210A variant [5]. Abnormal homocysteine metabolism and selected genetic polymorphisms may also be encountered in patients with thrombotic disease [6,7]. The contribution of these abnormalities to arterial thrombosis is not uniform. Although some can promote thrombosis through disturbances in anticoagulant pathways, endothelial function, platelet activity, or coagulation, the association of inherited thrombophilia with venous thromboembolism (VTE) is generally better established than its relationship with arterial thrombosis [5-7].

APS deserves particular consideration when thrombosis involves more than one vascular territory because it is an acquired disorder capable of producing both arterial and venous thrombotic events [8,9]. Therefore, the combination of ischemic stroke and ALI may warrant targeted evaluation for APS and other thrombophilic conditions, particularly when thrombosis occurs at a younger age, recurs, or cannot be adequately accounted for by established cardiovascular or structural vascular causes. Establishing a clinically relevant thrombophilic disorder may also influence decisions regarding secondary prevention and, in selected circumstances, the duration or type of antithrombotic therapy [8,9].

The prevalence and clinical profile of thrombophilia among patients who simultaneously develop ALI and ischemic stroke remain insufficiently characterized, with particularly limited evidence from resource-constrained healthcare settings. Previous investigations have predominantly focused on thrombophilia in patients with isolated ischemic stroke, VTE, or other arterial thrombotic conditions [10-12]. Consequently, there is limited information regarding which clinical features may help identify patients with this combined arterial presentation who are most likely to benefit from thrombophilia testing.

This study therefore assessed the prevalence and spectrum of selected inherited and acquired hypercoagulable abnormalities among adults presenting with concomitant ALI and ischemic stroke. It also examined demographic and clinical characteristics associated with the presence of a hypercoagulable disorder in this population.

## Materials and methods

Study design and setting

This prospective, hospital-based cross-sectional observational study was conducted in the Department of Neurology of a tertiary care teaching hospital in Bangladesh from January 2022 to December 2025. The study was designed to describe the prevalence and spectrum of selected inherited and acquired hypercoagulable abnormalities among adults presenting with concomitant ALI and imaging-confirmed ischemic stroke and to identify clinical characteristics associated with a hypercoagulable state.

Ethical approval was obtained from the Institutional Review Board of the participating institution (approval no. ERC-DMC/ECC/241/ 2021). Written informed consent was obtained from all participants or, when clinically necessary because of neurological impairment, from legally authorized representatives. The study was conducted in accordance with the Declaration of Helsinki.

Study population and participant selection

Adults aged ≥18 years presenting with both ALI and ischemic stroke during the study period were screened consecutively. Consecutive enrollment was used to minimize selection bias.

ALI was defined as an acute reduction in limb arterial perfusion with symptom duration of <14 days and clinical findings compatible with ALI. The diagnosis was established clinically and supported by objective vascular imaging, including duplex ultrasonography or computed tomography angiography (CTA), according to clinical circumstances. Limb ischemia severity was categorized according to the Rutherford classification as class I, IIa, IIb, or III [13].

Ischemic stroke was defined as an acute focal neurological deficit attributable to cerebral ischemia and confirmed by brain computed tomography (CT) or magnetic resonance imaging (MRI).

Patients with a definite alternative explanation for the concomitant arterial events, including traumatic arterial injury or iatrogenic arterial thrombosis following a vascular procedure, were excluded. Patients with a documented isolated cardioembolic source, such as atrial fibrillation with a convincing embolic mechanism or intracardiac thrombus, were excluded when this provided a sufficient explanation for both arterial events. This approach was used to enrich the study population for patients in whom an underlying thrombotic or hypercoagulable mechanism warranted evaluation.

Inclusion criteria

Patients were eligible if they were aged ≥18 years, had objectively confirmed ALI with symptom duration <14 days, had imaging-confirmed ischemic stroke occurring simultaneously or during the same hospitalization, underwent evaluation for selected inherited and acquired hypercoagulable disorders, and provided written informed consent or had consent from a legally authorized representative when appropriate.

Exclusion criteria

Patients were excluded if they had hemorrhagic stroke, traumatic arterial injury, iatrogenic arterial thrombosis after a vascular procedure, active malignancy, pregnancy, severe hepatic failure, active systemic infection or sepsis, incomplete thrombophilia investigations, or refusal to participate. Patients with a documented isolated cardioembolic source attributable to atrial fibrillation or intracardiac thrombus were also excluded when thrombophilia evaluation was not performed, because the study population was restricted to patients who underwent assessment for an underlying hypercoagulable state.

Definition of hypercoagulable or prothrombotic abnormality

For the primary analysis, a selected hypercoagulable or prothrombotic abnormality was defined as the identification of at least one predefined thrombophilic disorder or clinically relevant prothrombotic laboratory abnormality during the study evaluation.

Established thrombophilic disorders included APS, protein C deficiency, protein S deficiency, antithrombin deficiency, Factor V Leiden mutation, and prothrombin G20210A mutation. Hyperhomocysteinemia was considered a prothrombotic metabolic abnormality. Methylenetetrahydrofolate reductase (MTHFR) polymorphism was treated as an exploratory genetic finding and was not considered sufficient by itself to establish clinically significant thrombophilia.

Because individual abnormalities could coexist in the same patient, the frequencies of individual abnormalities were not mutually exclusive.

Sample size

The minimum sample size was calculated using the single-population proportion formula:

\[ n = \frac{Z^2 p(1-p)}{d^2} \]

where (n) is the required sample size, (Z) is the standard normal deviate for the selected confidence level, (p) is the anticipated prevalence, and (d) is the desired absolute precision.

Because a reliable local estimate of the prevalence of hypercoagulable disorders in patients with concomitant ALI and ischemic stroke was unavailable, (p=0.50) was used to provide the largest sample size for a proportion estimate. A 95% confidence level (Z=1.96) and absolute precision of (d=0.10) were selected. Thus,

\[ n = \frac{(1.96)^2 \times 0.50 \times (1-0.50)}{(0.10)^2} = 96.04 \]

At least 96 participants were therefore required. To accommodate possible exclusions and incomplete laboratory assessments, 100 consecutive eligible patients were enrolled.

Data collection

Data were collected prospectively using a standardized case-record form. Recorded variables included age, sex, body mass index (BMI), smoking status, hypertension, diabetes mellitus, dyslipidemia, coronary artery disease, chronic kidney disease, previous ischemic stroke, previous transient ischemic attack, previous VTE, recurrent arterial thrombosis, and family history of thrombosis.

Neurological severity at presentation was assessed using the National Institutes of Health Stroke Scale (NIHSS) [14]. ALI severity was categorized according to the Rutherford classification (classes I, IIa, IIb, and III). The affected limb, duration of ischemia, arterial level of occlusion, and available duplex or CTA findings were also documented.

Laboratory assessment of hypercoagulable disorders

After initial clinical stabilization, participants underwent laboratory evaluation for selected inherited and acquired thrombophilic abnormalities. The predefined thrombophilia evaluation included protein C activity, protein S activity, antithrombin activity, lupus anticoagulant, anticardiolipin antibodies (IgG and IgM), anti-β2-glycoprotein I antibodies (IgG and IgM), plasma homocysteine concentration, Factor V Leiden mutation analysis, and prothrombin G20210A mutation analysis.

Protein C, protein S, and antithrombin deficiency were defined according to the reference ranges and validated cutoffs of the testing laboratory. Because these assays may be influenced by acute thrombosis, anticoagulant therapy, liver dysfunction, or acute-phase responses, abnormal results were interpreted in the clinical context and, when clinically feasible, reassessed after the acute thrombotic phase.

APS was evaluated using lupus anticoagulant, anticardiolipin antibodies, and anti-β2-glycoprotein I antibodies. APS was classified according to the revised Sapporo/Sydney classification framework, requiring a qualifying thrombotic event together with persistent positivity for at least one relevant antiphospholipid antibody on two occasions separated by at least 12 weeks. Patients classified as having APS therefore had documented persistent antibody positivity.

Plasma homocysteine was assessed as a prothrombotic laboratory abnormality rather than automatically classified as an inherited thrombophilia. MTHFR polymorphism testing was performed selectively in patients with persistent hyperhomocysteinemia or a strong personal or family history suggestive of thrombophilia. MTHFR genotype was therefore considered an exploratory genetic finding and was not regarded as a definitive thrombophilic disorder in isolation.

Study outcomes

The primary outcome was the prevalence of any selected inherited or acquired hypercoagulable disorder among patients presenting with concomitant ALI and ischemic stroke. Secondary outcomes included the distribution of individual thrombophilic abnormalities, prevalence of APS, clinical characteristics associated with a hypercoagulable state, and independent clinical factors associated with the presence of a hypercoagulable disorder.

Statistical analysis

Statistical analysis was performed using IBM SPSS Statistics for Windows, Version 26 (Released 2019; IBM Corp., Armonk, New York, United States). Continuous variables were assessed for distribution using the Shapiro-Wilk test. Normally distributed continuous variables were summarized as mean ± standard deviation (SD), whereas non-normally distributed variables were summarized as median with interquartile range (IQR). Categorical variables were presented as frequencies and percentages.

Patients were categorized into two groups according to whether at least one selected hypercoagulable or prothrombotic abnormality was identified. Continuous variables were compared using the independent-samples Student's t test for normally distributed variables and the Mann-Whitney U test for non-normally distributed variables. Categorical variables were compared using Pearson's chi-square test or Fisher's exact test, as appropriate.

For the analysis of ALI severity, Rutherford classes IIb and III were combined as a predefined severe-ALI category. The association between Rutherford IIb/III ALI and the presence of a selected abnormality was assessed using logistic regression.

Univariable logistic regression was performed to identify demographic and clinical factors associated with the presence of a selected hypercoagulable or prothrombotic abnormality. Variables with p<0.20 in univariable analysis, together with clinically relevant covariates, were considered for the multivariable model. Age was categorized as <60 versus ≥60 years for the regression analysis. Results were expressed as odds ratios (ORs) and adjusted odds ratios (aORs) with corresponding 95% confidence intervals (CIs).

Multivariable logistic regression was subsequently performed to identify independent factors associated with the presence of a selected abnormality. Potential multicollinearity was assessed before final model construction. Model calibration was assessed using the Hosmer-Lemeshow goodness-of-fit test. All statistical tests were two-sided, and p<0.05 was considered statistically significant.

## Results

A total of 100 patients with concomitant ischemic stroke and ALI were included. Mean age was 56.8 ± 11.4 years (range, 24-81 years). Female subjects accounted for 62 of the participants, and the remaining 38 were male subjects. Hypertension was present in 69 patients, diabetes mellitus in 52, dyslipidemia in 45, and current smoking in 37. Previous ischemic stroke was reported in 34 patients and previous VTE in 18. The median NIHSS score was nine (interquartile range (IQR), 5-14). Rutherford class IIb /III ALI was present in 47 patients (Table 1).

**Table 1:** Baseline demographic and clinical characteristics of the study population (N=100) Data are presented as mean ± standard deviation (SD) for normally distributed continuous variables, median (IQR) for non-normally distributed continuous variables, and number (percentage) for categorical variables. SD, standard deviation; IQR, interquartile range; BMI, body mass index; NIHSS, National Institutes of Health Stroke Scale.

Variable	Value
Age (years), mean ± SD	56.8 ± 11.4
Female sex, n (%)	62 (62.0)
Male sex, n (%)	38 (38.0)
Body mass index (kg/m²), mean ± SD	25.2 ± 3.6
Current smoker, n (%)	37 (37.0)
Hypertension, n (%)	69 (69.0)
Diabetes mellitus, n (%)	52 (52.0)
Dyslipidemia, n (%)	45 (45.0)
Coronary artery disease, n (%)	29 (29.0)
Chronic kidney disease, n (%)	15 (15.0)
Previous ischemic stroke, n (%)	34 (34.0)
Previous venous thromboembolism, n (%)	18 (18.0)
NIHSS score, median (IQR)	9 (5–14)
Rutherford class IIb/III acute limb ischemia (ALI), n (%)	47 (47.0)

At least one hypercoagulable or prothrombotic abnormality was identified in 39 patients. APS was the most frequently identified abnormality, occurring in 18 patients, followed by hyperhomocysteinemia in 12, protein S deficiency in two, protein C deficiency in three, antithrombin III deficiency in two, Factor V Leiden mutation in three, and prothrombin G20210A mutation in one. All patients classified as having APS had a qualifying arterial thrombotic event and persistent antiphospholipid antibody positivity confirmed on repeat testing performed at least 12 weeks after the initial positive result. MTHFR polymorphism was identified in two patients and was recorded as an exploratory genetic finding. It was not considered a clinically significant thrombophilic abnormality when present in isolation and was therefore excluded from the definition of a hypercoagulable or prothrombotic state. Some patients had more than one abnormality; therefore, the individual abnormality frequencies were not mutually exclusive and their percentages were not expected to sum to the overall prevalence of 39 (Table 2). 

**Table 2:** Distribution of hypercoagulable disorders among the study population (N=100) Values are presented as a number (percentage). The prevalence of individual hypercoagulable disorders is shown for the overall study population. Because some patients had more than one hypercoagulable abnormality, the frequencies of individual disorders are not mutually exclusive, and the percentages do not sum to the overall prevalence of hypercoagulable states.

Hypercoagulable disorder	n (%)
Any hypercoagulable state	39 (39.0)
Antiphospholipid syndrome (APS)	18 (18.0)
Hyperhomocysteinemia	12 (12.0)
Protein S deficiency	2 (2.0)
Protein C deficiency	3 (3.0)
Antithrombin III deficiency	2 (2.0)
Factor V Leiden mutation	3 (3.0)
Prothrombin G20210A mutation	1 (1.0)

Patients with a hypercoagulable disorder were younger than those without an identified disorder (53.7 ± 9.8 vs. 58.8 ± 11.2 years; p=0.019). Female sex was more common in the hypercoagulable group (29/39 (74.4%) vs. 33/61 (54.1%); p=0.041). Previous ischemic stroke occurred in 18/39 (46.2%) patients with hypercoagulability compared with 16/61 (26.2%) without hypercoagulability (p=0.037). Previous VTE was present in 11/39 (28.2%) versus 7/61 (11.5%), respectively (p=0.031). Rutherford class IIb/III ALI was present in 27/39 (69.2%) patients with a hypercoagulable abnormality compared with 20/61 (32.8%) patients without a hypercoagulable abnormality (χ² = 13.07, p<0.001). Hypertension, diabetes mellitus, dyslipidemia, and current smoking did not differ significantly between groups (Table 3).

**Table 3:** Comparison of baseline clinical characteristics between patients with and without hypercoagulable disorders (N=100) Data are presented as mean ± standard deviation (SD) for continuous variables and number (percentage) for categorical variables. Continuous variables were compared using the independent-samples Student's t-test, whereas categorical variables were compared using the Pearson chi-square (χ²) test. Fisher's exact test was used when the expected cell count was <5. A two-sided p-value <0.05 was considered statistically significant. SD, standard deviation; χ², chi-square statistic; t, Student's t-statistic.

Variable	Hypercoagulable (n = 39)	Non-hypercoagulable (n = 61)	Test statistic	p-value
Age (years), mean ± SD	53.7 ± 9.8	58.8 ± 11.2	t = -2.40	0.019
Female sex, n (%)	29 (74.4)	33 (54.1)	χ² = 4.18	0.041
Hypertension, n (%)	24 (61.5)	45 (73.8)	χ² = 1.66	0.2
Diabetes mellitus, n (%)	18 (46.2)	34 (55.7)	χ² = 0.88	0.35
Dyslipidemia, n (%)	15 (38.5)	30 (49.2)	χ² = 1.12	0.29
Current smoker, n (%)	13 (33.3)	24 (39.3)	χ² = 0.35	0.55
Previous ischemic stroke, n (%)	18 (46.2)	16 (26.2)	χ² = 4.35	0.037
Previous venous thromboembolism, n (%)	11 (28.2)	7 (11.5)	χ² = 4.66	0.031
Rutherford class IIb/III acute limb ischemia (ALI), n (%)	27 (69.2)	20 (32.8%)	χ²= 13.07	<0.001

Univariable logistic regression showed associations between hypercoagulable disorders and age <60 years, female sex, previous ischemic stroke, previous VTE, and Rutherford class IIb/III ALI. Age <60 years was associated with higher odds of a hypercoagulable disorder (OR, 3.12; 95% CI, 1.40-6.95; p=0.005). Female sex was also associated with higher odds (OR, 2.47; 95% CI, 1.07-5.69; p=0.034). Previous ischemic stroke (OR, 2.42; 95% CI, 1.08-5.42; p=0.032), previous VTE (OR, 3.02; 95% CI, 1.13-8.07; p=0.028), and Rutherford class IIb/III ALI (OR, 4.61; 95% CI, 1.94-10.95; p<0.001) were also associated with hypercoagulability. Smoking, diabetes mellitus, hypertension, and dyslipidemia were not statistically significant predictors in univariable analysis (Table 4).

**Table 4:** Univariable logistic regression analysis of factors associated with hypercoagulable disorders among patients with concomitant acute limb ischemia and ischemic stroke Univariate logistic regression analysis was performed to identify factors associated with the presence of hypercoagulable disorders among patients with acute limb ischemia. Results are presented as odds ratios (ORs) with 95% confidence intervals (CIs). The Wald chi-square (χ²) statistic was used to test the significance of each regression coefficient. A two-sided p-value <0.05 was considered statistically significant.

Variable	Odds Ratio (95% CI)	Wald χ²	p-value
Age <60 years	3.12 (1.40–6.95)	7.83	0.005
Female sex	2.47 (1.07–5.69)	4.5	0.034
Current smoking	0.77 (0.34–1.73)	0.4	0.53
Diabetes mellitus	0.68 (0.30–1.53)	0.87	0.35
Hypertension	0.57 (0.24–1.36)	1.58	0.21
Dyslipidemia	0.65 (0.29–1.48)	1.07	0.3
Previous ischemic stroke	2.42 (1.08–5.42)	4.61	0.032
Previous venous thromboembolism	3.02 (1.13–8.07)	4.84	0.028
Rutherford class IIb/III acute limb ischemia (ALI)	4.61 (1.94–10.95)	12.1	<0.001

After adjustment for potential confounding variables, four factors remained independently associated with a hypercoagulable disorder. Patients aged <60 years had higher odds of hypercoagulability (aOR, 2.84; 95% CI, 1.16-6.91; p=0.022). Female sex remained independently associated with hypercoagulability (aOR, 2.18; 95% CI, 1.01-4.74; p=0.047). Previous VTE showed the strongest association (aOR, 3.91; 95% CI, 1.29-11.84; p=0.016).Rutherford class IIb/III ALI was strongly associated with the presence of a selected hypercoagulable or prothrombotic abnormality. It was present in 27 of 39 (69.2%) patients in the hypercoagulable group compared with 20 of 61 (32.8%) patients in the non-hypercoagulable group (OR, 2.86; 95% CI, 1.15-7.10; p=0.024). Previous ischemic stroke did not remain statistically significant after adjustment (Table 5).

**Table 5:** Multivariable logistic regression analysis of independent factors associated with hypercoagulable disorders in patients with acute limb ischemia and ischemic stroke (N=100) Multivariable logistic regression was performed to identify factors independently associated with the presence of a hypercoagulable disorder. Variables with p<0.20 in univariable analysis, together with clinically relevant covariates, were considered for inclusion in the multivariable model. Results are presented as adjusted odds ratios (aORs) with 95% confidence intervals (CIs). A two-sided p-value <0.05 was considered statistically significant.

Variable	Adjusted OR (95% CI)	Wald χ²	p-value
Age <60 years	2.84 (1.16–6.91)	5.23	0.022
Female sex	2.18 (1.01–4.74)	3.95	0.047
Previous venous thromboembolism	3.91 (1.29–11.84)	5.83	0.016
Rutherford class IIb/III acute limb ischemia (ALI)	2.86 (1.15–7.10)	5.1	0.024

## Discussion

This study evaluated selected thrombophilic abnormalities among adults who experienced acute ischemic events involving both the limb and the brain. At least one hypercoagulable disorder was detected in 39 of the 100 patients. APS was the most frequently identified abnormality, followed by hyperhomocysteinemia. Younger age (<60 years), female sex, previous VTE, and Rutherford class IIb/III ALI were independently associated with the presence of a hypercoagulable disorder.

Because the study population was restricted to patients with arterial ischemic events occurring in two vascular territories, the observed prevalence should not be extrapolated to patients with isolated ALI or ischemic stroke. Nevertheless, the identification of at least one abnormality in 39 (39.0%) patients indicates that selected patients with this uncommon clinical presentation may benefit from targeted evaluation for an underlying thrombotic predisposition. This observation is in keeping with recommendations favoring selective thrombophilia testing rather than routine screening, particularly in patients with unusual or recurrent thrombosis, younger age at presentation, or involvement of both arterial and venous vascular beds [10-12].

APS was identified in 18 patients and represented the most frequent thrombophilic abnormality in this cohort. Its presence is clinically relevant because APS is associated with arterial as well as venous thrombosis and may involve multiple vascular territories [8,9]. Thus, concomitant ischemic stroke and ALI may provide a clinical context in which APS evaluation is particularly appropriate when no sufficiently convincing alternative mechanism is identified. In the present study, APS classification required persistent antiphospholipid antibody positivity documented on testing separated by at least 12 weeks, in accordance with the revised Sapporo/Sydney framework [15].

Hyperhomocysteinemia was present in 12 patients. Increased homocysteine concentrations have been linked with cerebrovascular disease and may arise from a range of nutritional, metabolic, renal, and genetic factors [16,17]. However, the current study did not investigate the underlying cause of elevated homocysteine in individual participants. Therefore, its presence should be interpreted as an observed laboratory abnormality rather than evidence that hyperhomocysteinemia directly produced the thrombotic events. Similarly, MTHFR polymorphism was detected in two patients, but the genetic finding alone should not be regarded as evidence of a clinically significant thrombophilic disorder without considering the accompanying biochemical and clinical findings.

The inherited abnormalities identified in this study were relatively infrequent. Protein S deficiency occurred in two patients, protein C deficiency in three, antithrombin III deficiency in two, Factor V Leiden mutation in three, and prothrombin G20210A mutation in one. The relatively low frequency of inherited abnormalities is consistent with the limited and less well-established association between inherited thrombophilia and arterial thrombotic disease compared with VTE [5-7]. Accordingly, comprehensive testing for every inherited thrombophilic abnormality may have limited practical value in resource-constrained settings. A focused approach guided by factors such as younger age, recurrent thrombosis, family history, and thrombosis affecting multiple vascular territories may provide greater clinical utility.

Patients younger than 60 years had higher adjusted odds of having a hypercoagulable disorder (aOR, 2.84; 95% CI, 1.16-6.91). In individuals who develop arterial thrombosis at a relatively young age, the shorter duration of exposure to conventional atherosclerotic risk factors may increase clinical suspicion for alternative thrombotic mechanisms. Previous research involving younger adults with ischemic stroke has likewise highlighted the potential contribution of hypercoagulable conditions and their possible underrecognition [18]. Nevertheless, the present findings do not indicate that younger age is itself a cause of thrombophilia. Rather, age may serve as a clinical marker identifying patients in whom nonatherosclerotic mechanisms warrant greater consideration.

Female sex was also independently associated with hypercoagulability (aOR, 2.18; 95% CI, 1.01-4.74). The relatively high occurrence of APS among women provides one possible clinical explanation for this observation [8,9]. However, the study did not investigate sex-specific biological mechanisms, and the association should not be attributed specifically to APS because the outcome comprised several different inherited and acquired abnormalities.

A previous history of VTE showed the strongest independent association with hypercoagulability (aOR, 3.91; 95% CI, 1.29-11.84). The occurrence of both venous and arterial thrombosis may raise suspicion of a systemic prothrombotic condition and therefore represents an important component of the patient's thrombotic history. These findings support careful assessment of previous thrombotic events when determining whether thrombophilia testing is clinically justified. Laboratory results should also be interpreted together with the patient's overall clinical history rather than considered in isolation [6,7,11].

Rutherford class IIb/III ALI was independently associated with the presence of a hypercoagulable disorder (aOR, 2.86; 95% CI, 1.15-7.10). Severe limb ischemia may be associated with a larger thrombotic burden, acute thrombosis superimposed on pre-existing arterial disease, or other processes producing extensive arterial obstruction. However, because of the cross-sectional nature of this study, the observed association cannot establish whether thrombophilia contributed to the severity of ALI or whether severe ischemia and hypercoagulability were related through other clinical factors.

The findings may be particularly useful when deciding which patients with concomitant arterial ischemic events warrant further thrombophilia evaluation. The 2024 American College of Cardiology/American Heart Association (ACC/AHA) guideline emphasizes systematic assessment of ALI and identification of the underlying mechanism of arterial occlusion [19]. Thrombophilia testing should therefore complement, rather than replace, evaluation for established causes such as atrial fibrillation, intracardiac thrombus, atherosclerotic disease, and other structural vascular abnormalities. In patients with an unusual thrombotic pattern, particularly those with concomitant arterial and venous events or arterial thrombosis at a younger age, targeted testing may provide additional clinically useful information [20,21].

Interpretation of the laboratory results also warrants caution. Acute thrombosis, anticoagulant exposure, and acute-phase changes can influence measurements of protein C, protein S, and antithrombin. Antiphospholipid antibodies may also be transient in some circumstances. For this reason, APS assessment included repeat testing, while other thrombophilia results were considered in relation to the clinical context. Despite these precautions, the timing of laboratory assessment remains a potential source of uncertainty, and the observed prevalence should not be regarded as a definitive estimate of lifelong thrombophilia.

Limitations

This study has several limitations. First, it was performed at a single tertiary care center and included only 100 patients, which may restrict the generalizability and precision of the estimates. Second, the cross-sectional design permits identification of associations but does not establish temporal or causal relationships between thrombophilic abnormalities and the thrombotic events. Third, some thrombophilia assays may be affected by acute thrombosis, anticoagulant therapy, or acute-phase responses despite efforts to interpret or repeat abnormal results appropriately. Fourth, MTHFR testing was selective rather than uniform, and the laboratory evaluation of individual inherited abnormalities may therefore not have been identical in every participant. Fifth, the number of patients with each individual abnormality was small, limiting disorder-specific statistical analyses. Finally, long-term outcomes such as recurrent thrombosis, limb salvage, neurological recovery, functional status, and mortality were not analyzed according to thrombophilia status. The findings should therefore be considered hypothesis-generating and should not be interpreted as evidence that the identified abnormalities caused the concomitant arterial events.

Future multicenter prospective studies with larger and more diverse populations should confirm the prevalence estimates and clinical predictors identified here. Longitudinal follow-up would also help determine whether particular thrombophilic abnormalities predict recurrent arterial or venous thrombosis, limb-related outcomes, neurological recovery, or mortality.

## Conclusions

In this hospital-based study of adults presenting with concomitant acute limb ischemia and ischemic stroke, selected hypercoagulable or prothrombotic abnormalities were identified in a substantial proportion of patients. APS was the most frequently identified abnormality. Younger age, female sex, previous VTE, and more severe limb ischemia were independently associated with the presence of a hypercoagulable disorder. These findings suggest that selected patients with concomitant arterial thrombotic events may benefit from targeted evaluation for underlying hypercoagulable or prothrombotic abnormalities. Multicenter prospective studies with standardized laboratory testing and longitudinal follow-up are warranted to validate these associations and determine their clinical and prognostic significance.
